# The Patient And family member Reactions to biomarker‐informed ADRD DiagnosEs or PARADE Study

**DOI:** 10.1002/alz.087760

**Published:** 2025-01-09

**Authors:** Megan G. Witbracht, Melissa L Knox, Jennifer H Lingler, Joshua D Grill

**Affiliations:** ^1^ Institute for Memory Impairments and Neurological Disorders, University of California, Irvine, Irvine, CA USA; ^2^ University of Pittsburgh Alzheimer’s Disease Research Center (ADRC), Pittsburgh, PA USA

## Abstract

**Background:**

Amyloid imaging biomarkers serve an increasingly important role in diagnosing Alzheimer’s disease and determining eligibility for treatment with new disease‐modifying therapies. Yet, psychological and behavioral reactions to receiving a biomarker informed diagnosis remain relatively unstudied, especially in diverse and underserved populations where the burden of disease is high and resources for support are often insufficient. We developed the Patient And family member Reactions to biomarker‐informed ADRD DiagnosEs (PARADE) Study to address two key gaps in our understanding: 1) the range and trajectory of psychological and behavioral responses to a biomarker informed diagnosis and 2) the support needs of these individuals and their families.

**Method:**

Leveraging the infrastructure of New IDEAS, a coverage with evidence development study of amyloid PET, we developed a longitudinal study to quantify responses to receiving an amyloid PET biomarker test as part of a clinical work up for a diagnosis of cognitive impairment. Patients enrolled in the New IDEAS study are referred through the Alzheimer’s Association’s TrialMatch and then invited to participate with their study partners in 4 remote structured interviews performed prior to their amyloid PET scan and then 4‐, 12‐, and 24‐weeks after result disclosure. The primary outcome of the study is the Impact of Events Scale (IES), a measure of intrusive thoughts. Along with the IES, the interviews include questionnaires to assess the “value of knowing”, anxiety, depression, social support, loneliness, coping style, and discrimination. The goal of the study is to enroll 500 English or Spanish speaking dyads residing in the continental US. We will oversample Latinx and Black/African American participants as a reflection of the New IDEAS population.

**Result:**

Data collection began in May 2023 at the University of Pittsburgh and the University of California, Irvine and is expected to be complete the fourth quarter of 2026. Interviews have been completed by telephone and video conference in Spanish and English with relatively low rates of attrition.

**Conclusion:**

As the field advances to improve diagnosis and treatment, data from studies like this one will be essential to better understanding the patient and family experience of receiving a biomarker informed diagnosis.

